# Staged Flexible Transurethral Lithotripsy After Robot-Assisted Partial Nephrectomy for Concomitant Renal Cell Carcinoma and Ipsilateral Nephrolithiasis: A Case Report

**DOI:** 10.7759/cureus.113898

**Published:** 2026-08-03

**Authors:** Sohei Iwagami, Hikaru Nakao, Hiroyuki Koike

**Affiliations:** 1 Urology, Shingu Municipal Medical Center, Shingu, JPN; 2 Urology, Wakayama Medical University, Wakayama, JPN; 3 Urology, Shingu City National Health Insurance Kumanogawa Clinic, Shingu, JPN

**Keywords:** flexible transurethral lithotripsy, renal calculi, renal cell carcinoma, robot-assisted partial nephrectomy, treatment sequence

## Abstract

The optimal treatment sequence for patients with renal cell carcinoma (RCC) and concomitant ipsilateral renal calculi remains unclear. A 66-year-old man presented with a 45-mm left cystic renal mass adjacent to the collecting system and a 9-mm ipsilateral renal calculus. Because the stone was asymptomatic and oncological treatment was prioritized, robot-assisted partial nephrectomy (RAPN) was performed first. Minor collecting system entry occurred and was repaired intraoperatively. Histopathology revealed papillary RCC, pT3a, with negative surgical margins. Three months after RAPN, stone migration caused mild hydronephrosis without infection or urine leakage. Flexible transurethral lithotripsy (fTUL) was safely performed 4 months after RAPN using a low intrarenal pressure strategy. No perioperative complications occurred. Staged low-pressure fTUL after RAPN with collecting system repair may be a feasible treatment strategy in selected patients with concomitant RCC and ipsilateral renal calculi.

## Introduction

Partial nephrectomy (PN) is the standard treatment for localized renal cell carcinoma (RCC), and robot-assisted partial nephrectomy (RAPN) has become widely adopted in recent years [1]. Although patients with concomitant RCC and ipsilateral renal calculi are occasionally encountered in clinical practice, the optimal management strategy for patients presenting with both conditions remains unclear. Current major guidelines, including those of the European Association of Urology (EAU) and the American Urological Association (AUA), do not provide recommendations regarding the treatment sequence for concomitant RCC and ipsilateral renal stones [1-4].

Potential management strategies include stone treatment before RAPN, simultaneous treatment of both conditions, or staged endoscopic stone treatment after RAPN. Each approach has potential advantages and disadvantages with respect to oncological priority, urinary tract manipulation, collecting system healing, and the safety of subsequent endoscopic intervention. Because evidence comparing these strategies is scarce, treatment decisions are generally individualized in clinical practice.

Herein, we report a patient with a cT1b cystic RCC in close proximity to the collecting system and ipsilateral renal calculi who underwent oncologically prioritized RAPN followed by staged flexible transurethral lithotripsy (fTUL). This case may provide clinically relevant insights into treatment sequencing for patients with concomitant RCC and renal calculi requiring nephron-sparing surgery.

## Case presentation

A 66-year-old man was referred to our institution after a left renal mass was detected on abdominal ultrasonography during a routine health examination. His medical history was significant for hypertension, and renal function was preserved (Table 1).

**Table 1:** Laboratory tests. There were no abnormal values

Laboratory tests	Test values (normal range)
White blood cell count, 10^2^/μL	52 (33-86)
Hemoglobin, g/dL	15.5 (13.7-16.8)
Platelet, 10^4^/μL	23.5 (15.8-34.8)
Total Bilirubin, mg/dL	0.7 (0.4-1.5)
Aspartate aminotransferase, U/L	23 (13-30)
Alanine aminotransferase, U/L	34 (10-42)
Albumin, g/dL	4.3 (4.1-5.1)
Serum creatinine, mg/dL	0.9 (0.6-1.0)
Urinalysis: red blood cells, /HPF	0-1 (0-4)
Urinalysis: white blood cells, /HPF	0-1 (0-4)
Urine culture	Negative

**Figure 1:**
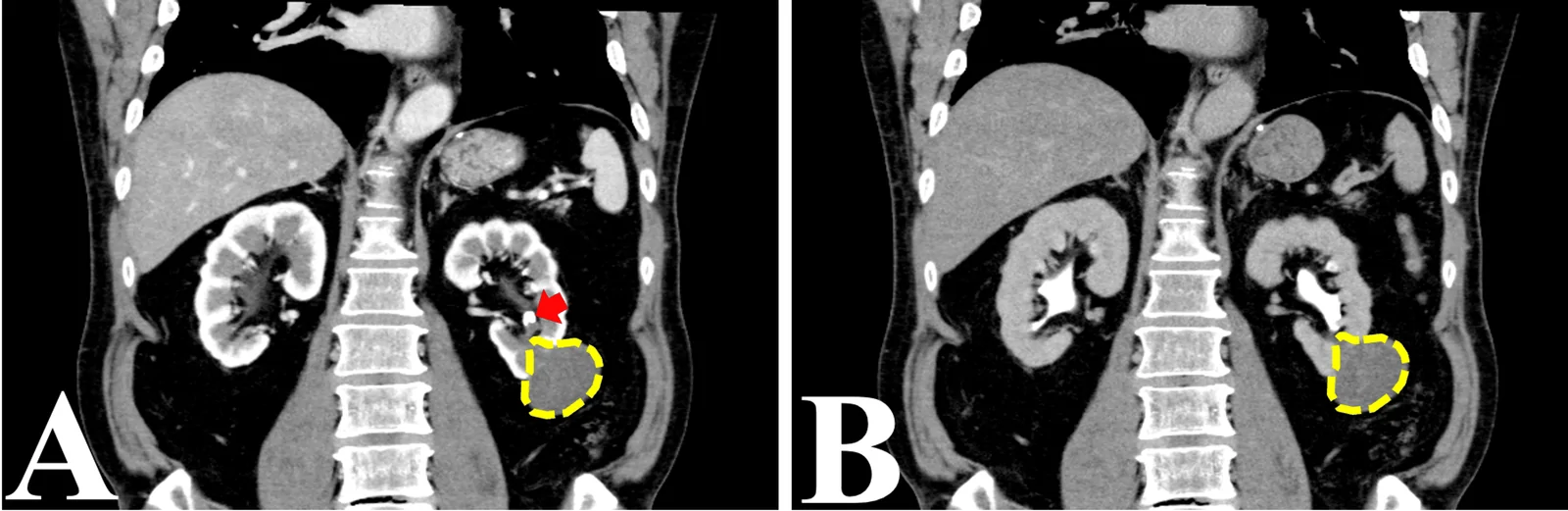
Preoperative imaging findings. (A) Contrast-enhanced computed tomography (equilibrium phase) demonstrated a 45-mm cystic renal mass (yellow dotted line) in the left lower pole, with partial protrusion toward the collecting system. An ipsilateral renal calculus is indicated by the red arrow. (B) Excretory-phase computed tomography demonstrated close proximity between the tumor (yellow dotted line) and the collecting system, with an estimated distance of approximately 3 mm. The R.E.N.A.L. nephrometry score was 7a (radius: 2, exophytic/endophytic: 1, nearness: 3, anterior/posterior: a, location: 1).

Contrast-enhanced computed tomography (CT) demonstrated a 45-mm cystic mass in the left lower pole (Figure 1).

**Figure 2:**
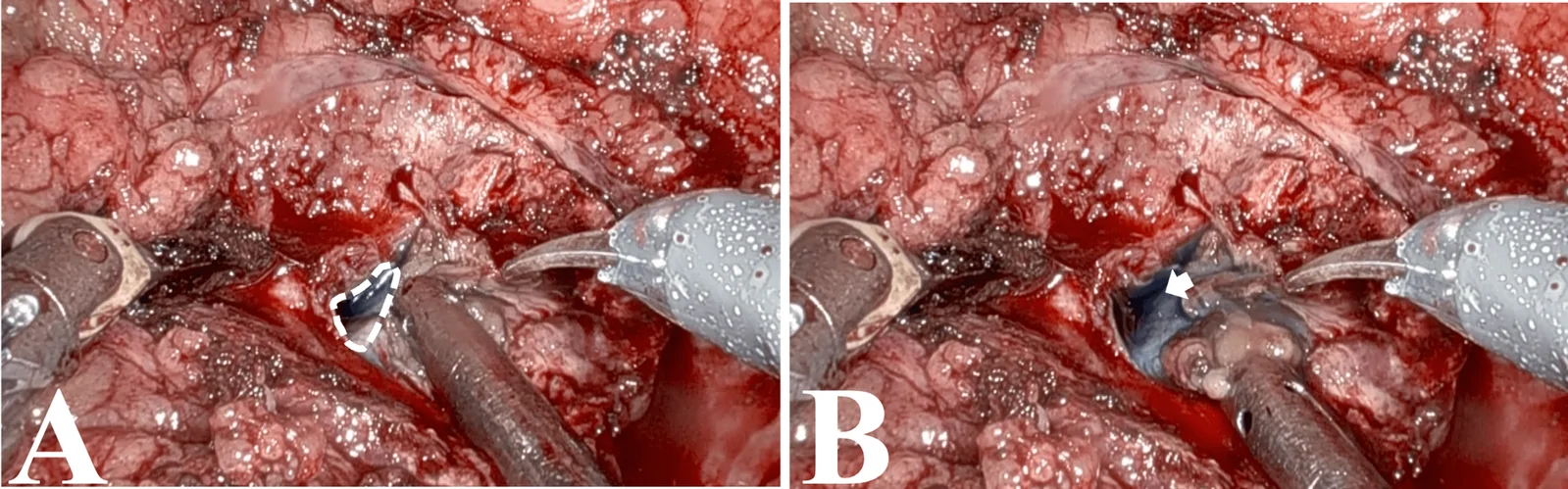
Intraoperative findings during robot-assisted partial nephrectomy. (A) Intraoperative view after tumor excision. The area surrounded by the white dotted line indicates collecting system entry. (B) Indigo carmine leakage from the collecting system confirmed the site of collecting system entry (white arrow).

The tumor protruded toward the collecting system and was closely adjacent to the urinary tract. In addition, a 9-mm radiopaque calculus was identified in the ipsilateral lower calyx. The patient was diagnosed with cT1bN0M0 RCC with ipsilateral nephrolithiasis. Because the stone was asymptomatic, RAPN was performed first. During RAPN, a minor collecting system entry occurred, which was repaired with primary suturing (Figure 2).

**Figure 3:**
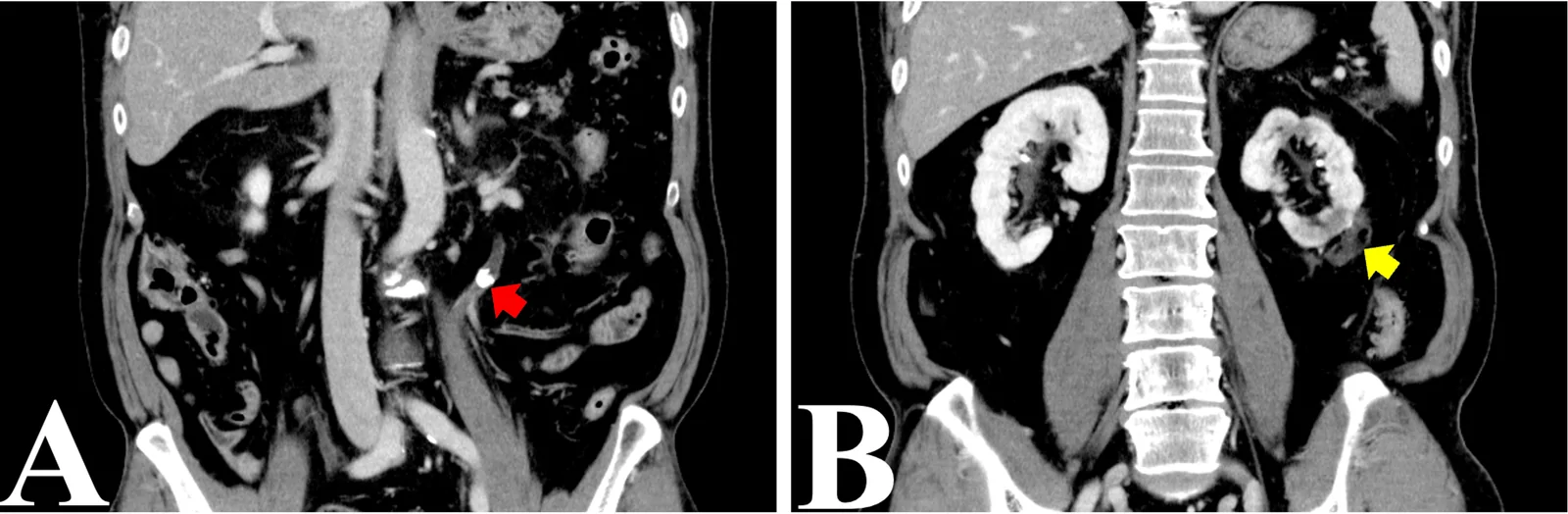
Postoperative computed tomography findings. (A) Computed tomography performed three months after robot-assisted partial nephrectomy demonstrated migration of the 9-mm calculus to the proximal ureter (red arrow). (B) Follow-up computed tomography showed no evidence of local recurrence, hemorrhage, urinary leakage, or hydronephrosis (yellow arrow).

Histopathological examination revealed papillary RCC, pathological stage pT3a with perirenal fat invasion and negative surgical margins. The postoperative course was uneventful. Three months after surgery, CT demonstrated migration of the stone into the proximal ureter with mild hydronephrosis (Figure 3).

**Figure 4:**
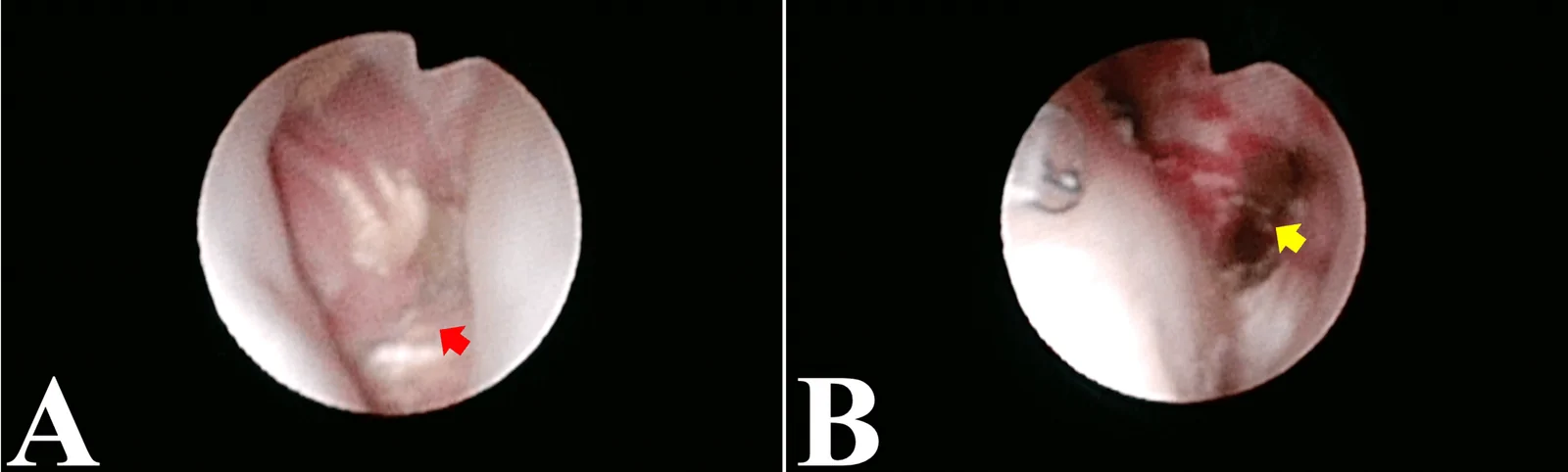
Intraoperative findings during flexible transurethral lithotripsy performed four months after robot-assisted partial nephrectomy. (A) Endoscopic view of the lower calyx corresponding to the previous site of collecting system entry. Although papillary loss was observed, the repaired collecting system appeared macroscopically healed without obvious defects (red arrow). (B) Endoscopic view during laser lithotripsy and stone extraction. Good visualization was maintained throughout the procedure (yellow arrow).

Because symptoms were minimal, conservative management was continued. As spontaneous stone passage did not occur, fTUL was performed one month later. The stone was relocated into the renal pelvis using a 6/7.5 Fr semi-rigid ureteroscope (Richard Wolf GmbH, Germany). A 10/12-Fr ureteral access sheath (Cook Medical, Bloomington, USA) was advanced to just below the ureteropelvic junction. Flexible ureteroscopy was performed using a URF-P7 flexible ureteroscope (Olympus Medical Systems Corp., Japan) under gravity irrigation (80 cmH₂O). Lithotripsy was carried out using a Pulse™ 30H Ho:YAG laser system (Lumenis Ltd., Israel) at 0.5-0.8 J and 5-8 Hz. The operative time was 50 minutes. No intraoperative complications occurred (Figure 4).

A 6-Fr × 24-cm Percuflex Plus™ ureteral stent (Boston Scientific, USA) was placed at the end of the procedure and removed four weeks later. Renal function remained stable throughout RAPN and staged fTUL. Subsequent follow-up CT confirmed stone-free status without postoperative complications. The patient remains under surveillance for oncological recurrence.

## Discussion

In the present case, RAPN was prioritized for a collecting system-adjacent cT1b RCC, followed by ipsilateral fTUL performed safely four months after surgery. No postoperative urine leakage, infection, or complications after staged fTUL occurred. Several reports have described flexible ureteroscopy after RAPN for postoperative complications such as clip migration; however, reports focusing on the treatment sequence for concomitant RCC and ipsilateral renal calculi after collecting system repair are extremely limited. This case demonstrates the feasibility of staged fTUL after RAPN with collecting system repair and provides insight into treatment sequencing for patients with concomitant RCC and ipsilateral renal calculi.

Compared with cT1a tumors, cT1b renal masses are associated with a higher risk of pathological upstaging and surgical complexity [5-9]. In the present case, the ipsilateral renal stone was asymptomatic and was not associated with infection or significant obstruction, whereas the 45-mm tumor was adjacent to the collecting system, and collecting system entry during RAPN was anticipated preoperatively. Therefore, oncological treatment was prioritized before stone treatment to achieve negative surgical margins and appropriate collecting system repair. Although pathological upstaging to pT3a could not be predicted preoperatively, the final pathological diagnosis supported the rationale for prioritizing oncological treatment in this patient.

Urine leakage after RAPN usually occurs early and resolves with conservative treatment or urinary drainage [10-12]. In the present case, at three months after RAPN, despite mild urinary tract obstruction caused by stone migration, no urine leakage or infectious complications developed, suggesting that the repaired collecting system had likely regained sufficient resistance to physiological urinary tract pressure. Urinary reconstruction generally requires several weeks to months to heal [13], supporting fTUL at four months after RAPN. Although direct evidence of complete healing of the repaired collecting system was not available, this case suggests that staged endoscopic stone treatment may be safely performed in clinically and radiologically stable patients.

Pyelorenal backflow may occur at pressures exceeding approximately 30-40 cmH₂O [14]. By contrast, the pressure threshold associated with collecting system rupture remains poorly defined, with experimental studies reporting values ranging from 70 to 400 cmH₂O [15]. In the present case, fTUL was performed using gravity irrigation at 80 cmH₂O and a 10/12 Fr ureteral access sheath (UAS) as part of a low intrarenal pressure strategy [16]. Rehman et al. reported that the use of a 10/12 Fr UAS reduced intrarenal pressure to approximately 22-29 cmH₂O during ureteroscopy [17]. Tokas et al. also emphasized the importance of maintaining low intrarenal pressure during retrograde intrarenal surgery to minimize pyelorenal backflow and infectious complications [14,18]. Although a direct causal relationship cannot be established, our findings suggest that, in addition to an adequate waiting period, a low intrarenal pressure strategy may contribute to the safe performance of staged fTUL after collecting system repair.

Although several reports have described ureteroscopic management of complications after partial nephrectomy [19,20], including urinary leakage and calyceal obstruction, evidence regarding staged fTUL after RAPN remains extremely limited. This report is limited by its nature as a single case, and therefore, the findings cannot be generalized. However, our experience suggests that staged fTUL after RAPN with collecting system repair can be performed safely in carefully selected patients. Further accumulation of cases is warranted to clarify the optimal timing and safety of endoscopic stone treatment after RAPN.

## Conclusions

This case demonstrates that staged flexible transurethral lithotripsy may be safely performed after robot-assisted partial nephrectomy with collecting system repair in carefully selected patients. When ipsilateral renal stones are asymptomatic and do not require urgent intervention, prioritizing oncological treatment followed by staged low-pressure endoscopic stone management after adequate postoperative assessment may represent a reasonable treatment strategy.
